# Evidence for Male Horn Dimorphism and Related Pronotal Shape Variation in *Copris lunaris* (Linnaeus, 1758) (Coleoptera: Scarabaeidae, Coprini)

**DOI:** 10.3390/insects9030108

**Published:** 2018-08-22

**Authors:** Kaan Kerman, Angela Roggero, Antonio Rolando, Claudia Palestrini

**Affiliations:** Department of Life Sciences and Systems Biology, University of Torino, Via Accademia Albertina 13, I-10123 Torino, Italy; kaan.kerman@edu.unito.it (K.K.); antonio.rolando@unito.it (A.R.); claudia.palestrini@unito.it (C.P.)

**Keywords:** dung beetles, cephalic horn, pronotal horn, geometric morphometrics, size, shape, allometry, size threshold, sneaker tactics, parental behavior

## Abstract

Male horn dimorphism is a rather common phenomenon in dung beetles, where some adult individuals have well-developed head horns (i.e., major males), while others exhibit diminished horn length (i.e., minor males). We focused on horn dimorphism and associated head and pronotum shape variations in *Copris lunaris*. We examined the allometric relationship between horn length (i.e., cephalic and pronotal horns) and maximum pronotum width (as index of body size) by fitting linear and sigmoidal models for both sexes. We then asked whether head and pronotum shape variations, quantified using the geometric morphometric approach, contributed to this allometric pattern. We found that female cephalic and pronotal horn growth showed a typical isometric scaling with body size. Horn length in males, however, exhibited sigmoidal allometry, where a certain threshold in body size separated males into two distinct morphs as majors and minors. Interestingly, we highlighted the same allometric patterns (i.e., isometric vs. sigmoidal models) by scaling horn lengths with pronotum shape, making evident that male horn dimorphism is not only a matter of body size. Furthermore, the analysis of shape showed that the three morphs had similar heads, but different pronota, major males showing a more expanded, rounded pronotum than minor males and females. These morphological differences in *C. lunaris* can ultimately have important functional consequences in the ecology of this species, which should be explored in future work.

## 1. Introduction

Male dimorphism—where some males possess well-developed, metabolically costly secondary sexual traits, while others retain diminutive structures to conserve valuable energy reserves—can be found in a diverse array of arthropod groups [1,2,3,4,5]. Beetle horns are a classic example of this type of environmentally controlled polyphenic trait [1,6,7]. In most dung beetle species (Coleoptera, Scarabaeidae), males with larger body sizes tend to support bulkier horns on their head and pronotum (i.e., majors). At the same time, smaller males either have diminished or no horn growth (i.e., minors). This unique pattern of male morphological differentiation is governed by early life conditions, such as the nutritional state of the developing larvae [8,9,10], and the quality of the parental investment received [11,12]. It ultimately plays a crucial role as alternative reproductive tactics in breeding [13,14]. The occurrence of dimorphic males is closely associated with distinct mating strategies across *Onthophagus* beetles, where major males defend key reproductive resources while minor males employ “sneaker” tactics to acquire reproductive benefits [15,16,17].

In the dung beetle species of the genus *Copris* Geoffroy, 1762, the presence of weapon-like projections on head is recurrent, but the occurrence of differential horn dimorphism has not yet been fully understood. In the speciose genus *Copris*, more than 250 species have been described so far. However, to our knowledge, only six *Copris* species have been investigated for horn dimorphism: *Copris ochus* Motschulsky, 1860; *C. acutidens* Motschulsky, 1860; *C. lugubris* Boheman, 1858; *C. klugi* Harold, 1869; *C. sierrensis* Matthews, 1961; and *C. armatus* Harold 1869. Horn dimorphism was highlighted in all the aforementioned species, starting from a study conducted on *C. lugubris* [2]. The scaling relationship between body size and cephalic horn length in *C. ochus*, *C. armatus*, *C. kluge*, and *C. sierrensis* differed between sexes, showing male horn dimorphism [18,19]. Similar results were obtained for both cephalic and pronotal horns in *C. acutidens*, suggesting that, for this species, the common developmental threshold mechanisms behind the emergence cephalic horn dimorphism could be involved in the growth of pronotal horns as well [20].

In *C. lunaris* (Linnaeus, 1758) large males have long head horns whereas small males and females do not. This species is relatively widespread in Palaearctic temperate areas ranging from Western Europe to China [21,22,23]. Although the reproductive behavior of this species, characterized by protracted female parental cares, is one of the most extensively studied in the genus [24,25,26], to our knowledge, no direct evidence for male dimorphism and alternative reproductive tactics has been presented, so far, in the literature.

The main objective of this paper was to document male horn dimorphism, if any, in this dung beetle species, since finding male horn morphs in *C. lunaris* might be a clue of alternative reproductive tactics in this species. We also focused on females, assuming their complex parental behavior could reverberate on some morphological traits. Our secondary objective was to investigate the developmental connection between the horns and other prominent body parts, such as head and pronotum. Prior works on another dung beetle group (genus *Onthophagus*) has shown that shape modifications in head and pronotum can be associated with the relative size of horn structures in males [27,28]. Nevertheless, this topic of how eventual dimorphic males and females vary in both head and pronotum shape is yet to be explored in the genus *Copris*. In this study, we investigated the allometric relationship between horn length and body size using the traditional, linear morphometric measures to detect horn allometry in males and females. We then applied the geometric morphometric techniques to assess whether shape variations in head and pronotum were correlated with distinct morphs.

## 2. Materials and Methods

### 2.1. Material

We performed morphological analyses on a total of 76 Western Palaearctic specimens (52 males and 24 females), given on loan by the Museo Civico di Storia Naturale, Milano, Italy (MSNM) and Museo Civico di Storia Naturale, Carmagnola, TO, Italy (MCCI).

### 2.2. Data Acquisition

We captured the 2D images of head and pronotum by the software LAS-Leica Application Suite (Leica Microsystems AG, Wetzler, Germany), using a Leica^®^ DMC4500 (Leica Microsystems AG, Wetzler, Germany) digital camera connected to a stereoscopic dissecting scope Leica^®^ Z16APO (Leica Microsystems AG, Wetzler, Germany), having care to avoid the malpositioning of the specimens.

MicroCT non-invasive 3D techniques [29,30,31] were applied to evaluate cephalic and pronotal horn structures of *C. lunaris*, in keeping with the methods employed in various arthropod taxa [32,33,34,35,36,37,38]. The scans were performed by a Bruker^®^ SkyScan 1174 (Bruker microCT, Kontich, Belgium), using the Bruker SkyScan (Bruker microCT, Kontich, Belgium) software series (i.e, SkyScan 1174v2 control software v1.1, NRecon v1.7.1.0, Data Viewer v1.5.2.4, and CTVox v3.3.0) for the data acquisition and reconstruction. Scans were done applying the following parameters: image rotation = 0.36°; source voltage = 34 kV; source current = 793 μA; image pixel size = 14.10 or 16.71 μm; exposure = 2100 ms; rotation step = 0.1°; 360° rotation = OFF; frame averaging = 2; sharpening = 40%; filter = OFF. Each scan took about 4 h duration, on average.

### 2.3. Morphometric Measurements

#### 2.3.1. Horn Length

*Copris lunaris* males possess a long recurved cephalic horn, a carina-like anteromedial prominence that extends from the center of the pronotum, and two symmetrical lateral prominences on the pronotum [39]. We focused on the cephalic horn and on the carina extending from the center of the pronotum, hereafter called, for the sake of simplicity, the pronotal horn.

**Figure 1:**
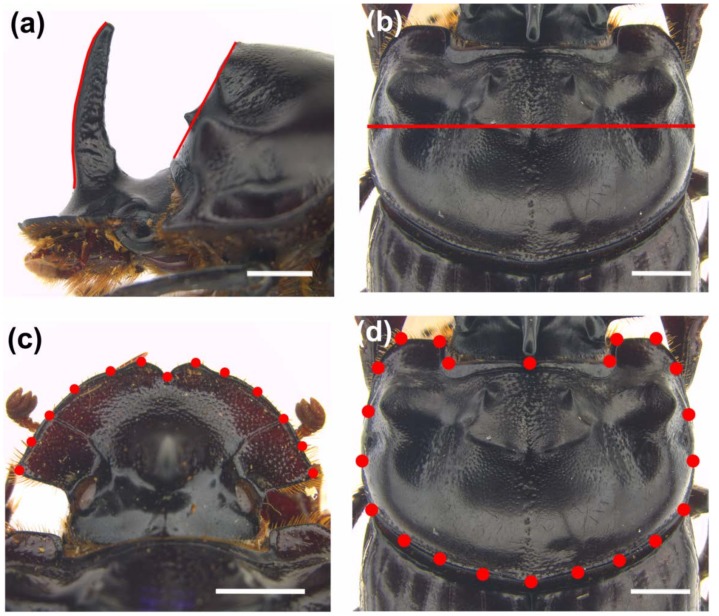
*Copris lunaris*. (**a**) Cephalic and pronotal horn length, and (**b**) maximum pronotum width measures; (**c**) head and (**d**) pronotum landmark configurations. Scalebars = 2 mm.

Cephalic horn length in *Copris* species has traditionally been quantified on the lateral view of the head as a straight line between the tip and base of the horn [2,18,20]. For a better representation of the horn curvature, we instead traced a curved line from the base to the tip of the horn (Figure 1a). This method was formerly applied with good results in other dung beetle species [19]. Pronotal horn length (Figure 1a) was measured as the length of the anterior declivity from the anterior margin of the pronotum to the dorsal tip of the pronotal carina, in accordance with former analyses [20]. All measures were captured by the LAS Measurement Module of the software Leica Application Suite (LAS), and were expressed in mm.

#### 2.3.2. Body Size

We estimated body size (expressed in mm) by calculating the maximum width of the pronotum as a linear morphometric measure [18,40], using the LAS software. The pronotum width (Figure 1b) is generally considered a reliable index of body size in coleopteran taxa [2,19,41,42].

#### 2.3.3. Shape

We applied the geometric morphometrics semilandmark-based approach [43,44,45,46,47,48,49] to describe the overall shape variation of head and pronotum. The software tpsDig v2.31 [50] and tpsUtil v1.76 [51] (http://life.bio.sunysb.edu/morph/) were used to define the point configurations (Figure 1c,d) of the head (N_H_ = 13) and pronotum (N_P_ = 22).

### 2.4. Analysis of Horn Allometry

We investigated the cephalic and pronotal horn allometries by looking at the scaling relationship between horn length and body size in males and females separately [28,42,52]. We evaluated the allometric lines by fitting at first a simple linear regression, then a non-linear function expressed by Hill’s sigmoidal curve. The model fitting was evaluated using the software PAST v3.20 (http://folk.uio.no/ohammer/past) [53], and SigmaPlot v10.0 (Systat Software Inc., San Jose California, CA USA, 2007).

In order to determine the model that best described the allometric relationship in each sex, we calculated Akaike information criterion (AIC) values for each model, i.e., linear (AIC_L_) and Hill’s sigmoid (AIC_S_), as an index of their goodness of fit [52,54]. Models with lower AIC scores were considered better fit to the dataset. In the case of an observed dimorphism, major and minor morphs were defined by the estimated switch point [28,55]. Individuals that grew larger than that switch point were considered major males, while smaller ones were grouped as minor males. Finally, we compared the pronotum width, cephalic and pronotal horn lengths across morphs using Kruskal–Wallis Test with Bonferroni correction (significance level = 0.05) in the statistical software SPSS v24 (IBM Corp., Armonk, NY, USA).

### 2.5. Analysis of Shape Variation

Following the morphs estimation, the overall shape variation for both head and pronotum was analyzed by principal component analysis (PCA) using tpsRelw v1.69 [56] (http://life.bio.sunysb.edu/morph/). The relative warp values (i.e., RWs) that cumulatively explained 100% of the overall shape variation for each anatomical structure were then employed in the canonical variate analysis (CVA) using IBM SPSS v24 to evaluate the group membership accuracy by means of the cross-validation.

### 2.6. Analysis of Size and Shape Covariation

To analyze the scaling relationships between horn lengths and pronotum shape across morphs, we compared the horn length values with pronotum RWs 1–3 (percent values of explained shape variation >5%) and evaluated the best fit correlation for size vs. shape [57] by PAST.

## 3. Results

### 3.1. Horn Allometry

**Figure 2:**
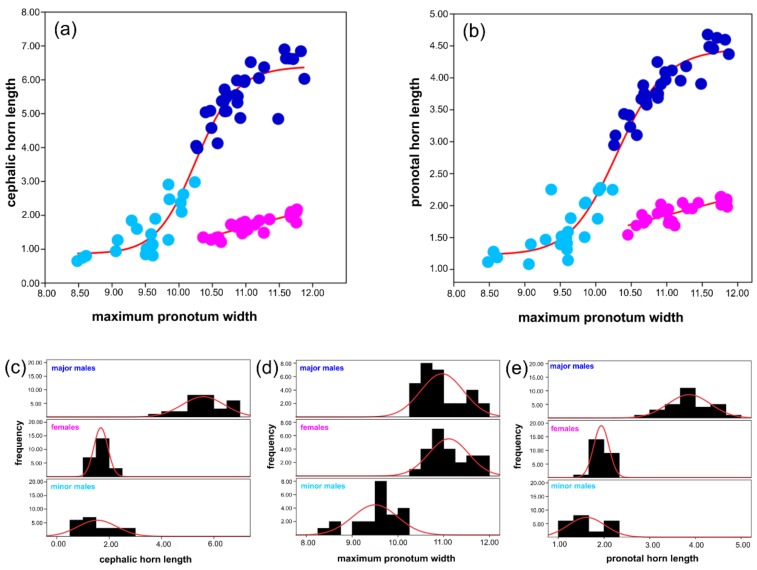
Horn allometry. (**a**) Scatterplot of the cephalic horn length and body size (i.e., maximum pronotum width). The best-fitting lines were showed on the plot for both sexes (red lines) separately, with the better AIC value being 18.285 for males (Hill’s sigmoid function) and 5.204 for females (linear function); (**b**) scatterplot of the pronotal horn length and body size (i.e., maximum pronotum width). The best-fitting lines were showed on the plot for both sexes (red lines) separately, with the better AIC value 11.775 being for males (Hill’s sigmoid function) and 4.807 for females (linear function). In both plots, the morphs were distinguished by dark blue (major males), light blue (minor males), and fuchsia (females) dots. Histograms of (**c**) cephalic horn length, (**d**) pronotum width, and (**e**) pronotal horn length frequency per morphs.

In females, the allometric lines produced by the linear model showed a better fit than the sigmoidal curve (Figure 2), indicating the presence of an isometric growth for both cephalic (AIC_L_ = 5.020, and AIC_S_ = 10.543) and pronotal (AIC_L_ = 4.807, and AIC_S_ = 10.331) horns.

Males, on the other hand, exhibited a sigmoidal pattern of horn growth (Figure 2) both for the cephalic (AIC_L_ = 32.631, and AIC_S_ = 18.285, switch point x = 10.394, y = 5.015) and pronotal (AIC_L_ = 12.11, and AIC_S_ = 11.775, switch point x = 10.394, y = 3.463) horns. Individuals larger than the switch point were classified as major males, while smaller ones were categorized as minor males.

Following the comparison of horn lengths across morphs (Table 1), we found that major males had longer cephalic horns than minors (H = 41.045, *p* < 0.05). Females supported horns that were significantly shorter than major males (H = 35.208, *p* < 0.05) while demonstrating similar horn length with minor males (H = 5.837, *p* = 0.371). Similar to cephalic horns, major males possessed more developed pronotal horns than minors (H = 43.591, *p* < 0.05) and females (H = 32.875, *p* < 0.05). We observed no difference between minor males and females in pronotal horn length (H = 10.716, *p* = 0.100). Finally, when we looked at pronotum width (i.e., body size) across morphs, we observed that major males were larger than minor males (H = 35.483; *p* < 0.05), while females showed similar body sizes with major males (H = −5.662, *p* = 0.349), and were considerably larger than minor males (H = 41.146, *p* < 0.05).

### 3.2. Shape Analysis

**Figure 3:**
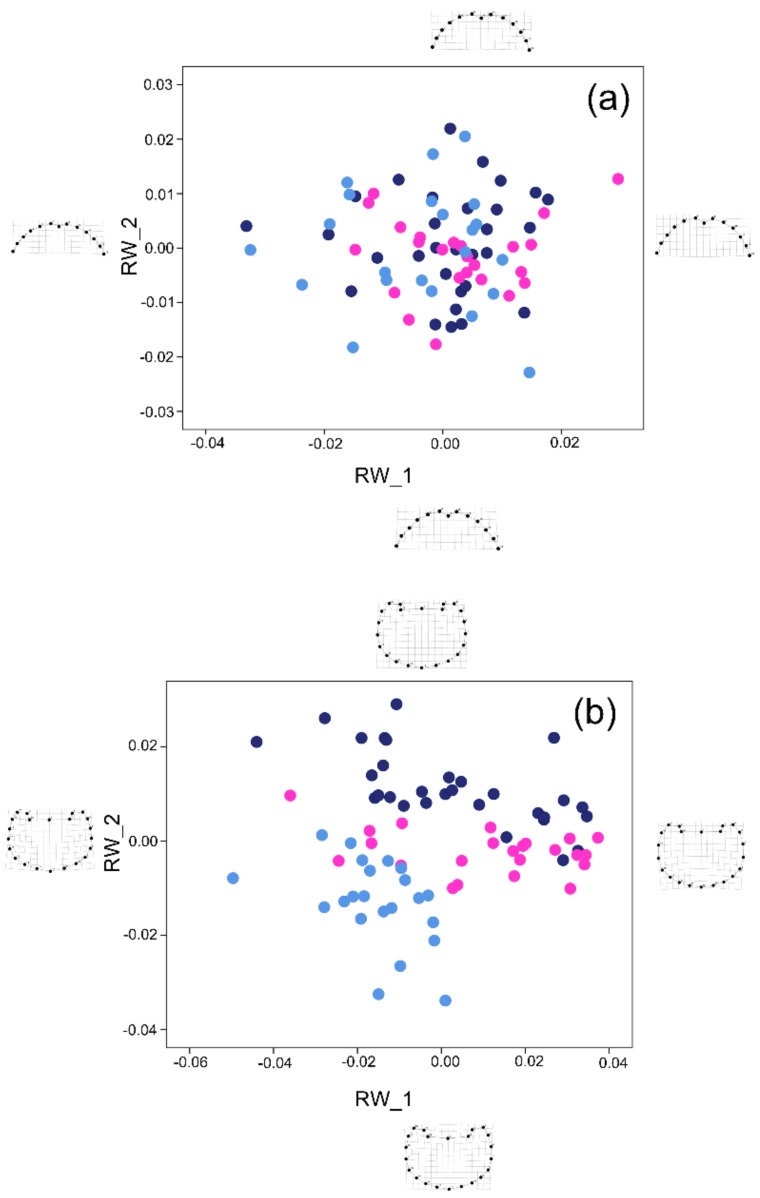
Shape analysis. Scatterplot of the RWs 1 and 2; morphs are distinguished by dark blue (major males), light blue (minor males), and fuchsia (females) dots. (**a**) Head, showing 59.58% of the overall shape variation; (**b**) pronotum, showing 66.54% of the overall shape variation. On both plots, the deformation grids of the two axes extremities are shown.

In the analysis of the head, 18 out of 22 RWs explained 100% of the overall shape variation, with the first two RWs explaining about the 60% (Figure 3a, 36.28% for RW_1, and 23.30 for RW_2). Visual inspection of head shape differences in the scatterplot revealed an overlap between all three groups (i.e., major males, minor males, and females) with no clear divergence pattern (Figure 3a). The CVA results (Table 2) corroborated the marked homogeneity of head shape variation in the three morphs (only 63.2% of cross-validated grouped cases were correctly classified).

In the analysis of the pronotum, 30 out of 40 RWs explained 100% of the overall shape variation, with the first two RWs explaining about 67%. As opposed to head shape, pronotum shape differed notably between the groups, forming distinct clusters in the scatterplot (Figure 3b, 48.86% for RW_1, and 17.69% for RW_2, respectively). Deformation grids of both RW_1 and RW_2 showed that shape variation especially concerned the anterior part of the pronotum (far more marked in the RW_2 grids), as major males had a more expanded, rounded pronotum than minor males and females.

The CVA results (Table 2) confirmed the separation of the morphs, with 89.3% of the cross-validated grouped cases correctly classified.

### 3.3. Shape vs. Size Analysis

**Figure 4:**
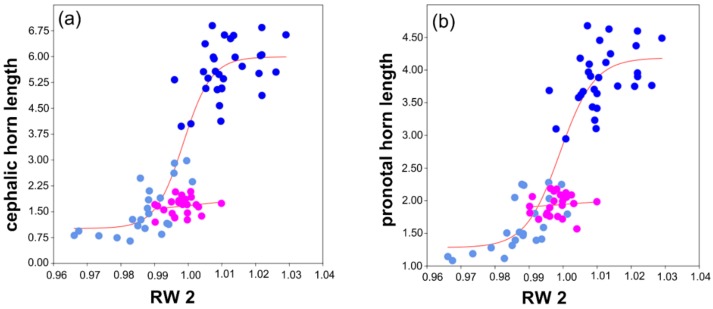
Size vs. shape analysis. (**a**) Scatterplot of the RW_2 and cephalic horn length with the best fit line, with the AIC value being 40.570 (Hill’s sigmoid function) for males, and 5.947 (linear function) for females. (**b**) Scatterplot of the RW_2 and pronotal horn length with the best fit line, with the AIC value being 19.895 (Hill’s sigmoid function) for males, and 5.227 (linear function) for females. In both plots, the morphs are distinguished by dark blue (major males), light blue (minor males) and fuchsia (females) dots.

To analyze the relationships between horn lengths and pronotum shape across morphs, we compared the horn length values with pronotum RWs 1–3. When scaling cephalic and pronotal horn length with RW_1 and RW_3, major males were well separated from minor males and females, but no function could be fitted to data distributions to explain the relationships among the morphs. However, when scaling both cephalic and pronotal horn length with RW_2, all the three morphs were well separated, furthermore, the Hill’s sigmoid function could be fitted for male morphs and the linear one for females (Figure 4).

### 3.4. MicroCT Images

**Figure 5:**
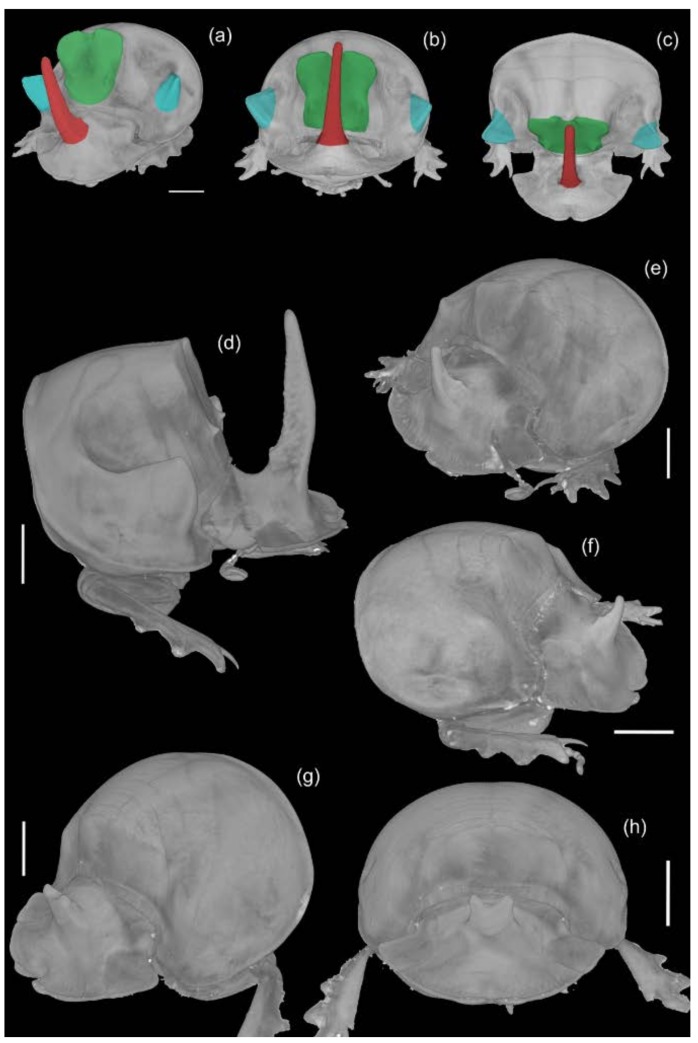
MicroCT images of the head and pronotum of the *C. lunaris* morphs. (**a**) Major male, with the various parts marked by colors: head horn (red), lateral pronotal prominences (blue), and pronotal horn (green); major male, the horn positioned above the median groove, (**b**) anterior view, and (**c**) dorsal view; (**d**) major male, with exaggerated development of the head and pronotum; (**e**,**f**) minor males, with different development of the pronotum; female, (**g**) side view, and (**h**) frontal view. Scalebars = 2 mm.

The microCT non-invasive 3D technique revealed the complexity of the head and pronotal morphology. Major males supported a long cephalic horn, a prominent pronotal carina (i.e., the pronotal horn) and two lateral pronotal prominences; they also had a deep groove along the central carina of their pronotum. Minor males and females did not have such exaggerated development of the head and pronotum (Figure 5).

## 4. Discussion

The males of *C. lunaris* exhibit a double horn (both cephalic and pronotal) dimorphism, while females show a linear growth pattern. More in detail, female cephalic and pronotal horn growth showed a typical isometric scaling with body size. Horn length in males, however, exhibited sigmoidal allometry, where a certain threshold in body size (and horn length) separated males into two distinct morphs as majors and minors. Interestingly, we highlighted the same allometric patterns (i.e., isometric in females vs. sigmoidal in males) by scaling horn lengths with pronotum shape (relative warp 2). This result formally demonstrates that male dimorphism is not only a matter of body size because horn growth is also associated to variations in the shape of the pronotum. Overall, major males had more expanded and rounded pronotum, and supported longer cephalic and pronotal horns than minors.

Although in some other *Copris* species [18], a marked sexual dimorphism in body size was highlighted, also with smaller females, here, the females are always larger than minor males, and as large as major males. The results suggested a possibility that small females do not exist in *C. lunaris*, or they could be extremely rare, thus, the female dataset could be biased towards larger females, and errors caused by this bias should be taken into account.

Our results support the general consensus of horn dimorphism in the genus *Copris.* Presence of cephalic horn dimorphism in males was well documented in the *Copris* species studied thus far [2,18,19,20]. Some members of the genus even exhibit pronotal horn dimorphism in addition to the more commonly known cephalic horn dimorphism, where major males possess longer horns coupled with increased horn height from the dorsal surface of the body [19,20].

Interestingly, head shape remained independent of dimorphic grouping of males, as well as between males and females. At the same time, pronotal shape exhibited marked differences across groups. This is in contrast to other dung beetle taxa such as *Onthophagus*, where male morphs can exhibit marked differences both in head and pronotum morphology [27,28]. Although it is not clear why dimorphic males resemble each other in their head shape patterns, anatomical structures, such as horns, can certainly develop in tandem with more distant body parts if there is a functional aspect involved [58,59]. It is likely that these pronotal shape differences are functionally related to intrasexual competition among *C. lunaris* males during the breeding season.

At the onset of the breeding period, both sexes excavate underground tunnels and transfer large amounts of dung to brood chambers [25,26,60]. Once brood balls are constructed, females initiate their extended brood care by sealing themselves off inside the brood chamber until their larvae emerge as adults. Before the sealing of brood chambers by females, courting males position themselves at tunnel entrances close to the surface to fend off potential male intruders. It has been suggested that larger *C. lunaris* males do not use their horns in outright fights, but implement them to block tunnel entrances [26], probably against smaller, more agile “sneaker” males. Major males that support large pronotum with narrow frontal angle, coupled with longer cephalic and pronotal horns, might be more successful at blocking tunnel entrances against intruders.

Horn dimorphism in *C. lunaris* can have long term ecological consequences. *Copris lunaris* significantly contributes to methane (CH_4_) release from dung pats in contrast to other common but smaller dung beetle species across Europe [60]. This could be a direct result of the extended maternal care over brood balls, which, in turn, keeps dungs fresh for longer duration. Presence of females in brood chambers facilitates the guarding behavior of major males at the tunnel entrances. Also, major males could help breeding females in transferring dung into brood chambers. Although we do not know exactly how major and minors differ in their contribution during dung removal in this species, we suspect that large males were the main cooperators during nest building. This could mean that a shift in dimorphic male ratios towards minor males due to environmental factors can have significant impact on their ecosystem services.

Finally, we suggest these differences may be explained by multiple factors. (1) Exaggeration in no sexual traits may accompany exaggeration in the primary targets of sexually selected traits [61], leading to coupling of exaggerated traits and correlated modifications in other somatic characters [62]. This idea may help us to understand the differences of pronotum. (2) Differences between minor males and females may also be the result of sexual dimorphism, whereas (3) differences between males may be functionally related to intrasexual competition among males during the breeding season [63]. (4) Lastly, we have found that major males have a deep groove along the central carina of their pronotum, which may accommodate their cephalic horns when males place their heads back during confrontations. This accommodation suggests that cephalic and pronotal structures may also develop in concert.

## 5. Conclusions

In conclusion, the presence of an underlying social complexity during reproduction for this group of dung beetles (i.e., *Copris*), from alternative reproductive tactics to acoustic signaling between breeding pairs and larvae [64,65], necessitates a detailed investigation into their ecological context. We believe future work on different dimorphic male compositions in natural settings, together with a more detailed look at morphological differences in other body parts, could help us understand the development, ecology, and evolution of the reproductive behavior of this species.

## Figures and Tables

**Table 1:** Descriptive statistics of the cephalic and pronotal horn length, and pronotum width (body size). Measures were taken in mm.

		Mean ± SE		
	*n*	Cephalic Horn Length	Pronotal Horn Length	Max Pronotum Width
females	24	1.68 ± 0.27	1.94 ± 0.17	11.11 ± 0.43
major males	30	5.59 ± 0.80	3.86 ± 0.46	10.96 ± 0.47
minor males	22	1.55 ± 0.74	1.61 ± 0.40	9.50 ± 0.49

**Table 2:** Canonical variate analysis (CVA) classification results for head and pronotum. The cross-validated values are given for each morph (1 = major males, 2 = females, 3 = minor males). For the head, 63.2% of the cross-validated grouped cases were correctly classified, and for the pronotum, 89.3% of the cross-validated grouped cases were correctly classified.

Classification Cross-Validated Results						
			predicted group membership			total
			1	2	3	
head	count	1	19	6	5	30
		2	5	14	5	24
		3	4	3	15	22
	%	1	63.3	20.0	16.7	100
		2	20.8	58.3	20.8	100
		3	18.2	13.6	68.2	100
			predicted group membership			total
			1	2	3	
pronotum	count	1	25	5	0	30
		2	0	21	2	23
		3	0	1	21	22
	%	1	83.3	16.7	0.0	100
		2	0.0	91.3	8.7	100
		3	0.0	4.5	95.5	100
